# Relationships Between Sport-Specific Anaerobic Tests, Interlimb Asymmetry, and Bilateral Deficit as Measured from Vertical Jump Performances in Highly Trained Taekwondo Athletes

**DOI:** 10.3390/sports13040103

**Published:** 2025-03-28

**Authors:** Ibrahim Ouergui, Slaheddine Delleli, Gennaro Apollaro, Hamdi Messaoudi, Rafael Lima Kons, Craig Alan Bridge, Hamdi Chtourou, Emerson Franchini, Emanuela Faelli, Luca Paolo Ardigò

**Affiliations:** 1 High Institute of Sport and Physical Education of Kef, University of Jendouba, El Kef 7100, Tunisia; 2 Research Unit, Sports Science, Health and Movement, University of Jendouba, El Kef 7100, Tunisia; 3 High Institute of Sport and Physical Education, University of Sfax, Sfax 3038, Tunisia; sdelleli2018@gmail.com (S.D.); hamdimessaoudihamdi@gmail.com (H.M.); hamdi.chtourou@isseps.usf.tn (H.C.); 4 Physical Activity, Sport, and Health, UR18JS01, National Observatory of Sport, Tunis 1003, Tunisia; 5 Department of Neuroscience, Rehabilitation, Ophthalmology, Genetics and Maternal Child Health, University of Genoa, 16132 Genoa, Italy; gennaro.apollaro@edu.unige.it; 6 Centro Polifunzionale di Scienze Motorie, University of Genoa, 16132 Genoa, Italy; emanuela.faelli@unige.it; 7 Department of Physical Education, Faculty of Education, Federal University of Bahia, Salvador 40170-110, Brazil; rafakons0310@gmail.com; 8 Sports Performance Research Group, Department of Sport & Physical Activity, Edge Hill University, Lancashire L39 4QP, UK; bridgec@edgehill.ac.uk; 9 Martial Arts and Combat Sports Research Group, Sport Department, School of Physical Education and Sport, University of São Paulo, Sao Paulo 05508-060, Brazil; efranchini@usp.br; 10 Department of Experimental Medicine, Section of Human Physiology, University of Genoa, 16132 Genoa, Italy; 11 Department of Teacher Education, NLA University College, Linstows Gate 3, 0166 Oslo, Norway

**Keywords:** asymmetry, performance, strength, correlation, combat sports

## Abstract

The present study investigated the effect of inter-limb asymmetry and bilateral indexes (BLIs) in power performance, assessed by squat jump (SJ) and countermovement jump (CMJ), on sport-specific anaerobic performance. Forty-eight taekwondo athletes (24 males and 24 females; mean ± SD, age: 19.8 ± 2.1 years) performed randomly, in separate sessions, SJ and CMJ tests using the right, left, and both legs to determine the asymmetry and BLI, the 10 s Frequency Speed of Kick Test (FSKT_10s_), and its multiple version (FSKT_mult_). FSKT_10s_ was positively correlated with CMJ asymmetry (ρ = 0.299, *p* = 0.039, low) and SJ BLI (r = 0.596, *p* < 0.001, large), and negatively correlated with CMJ BLI (r = −0.472, *p* = 0.001, moderate). Similarly, FSKT_mult_ was positively correlated with SJ BLI (ρ = 0.632, *p* < 0.001, large), and negatively correlated with CMJ BLI (ρ = −0.532, *p* < 0.001, large). CMJ and SJ BLI explained 45.9% of the variance of the FSKT_10s_ performance, whilst they explained 55.7% of the variance of FSKT_mult_ performance. These results highlight the importance of managing both interlimb asymmetry and bilateral deficit indexes to achieve better performance by improving the strength of the non-dominant leg and using exercises that are performed bilaterally despite unilateral movements being predominant in taekwondo, which can potentially enhance the effectiveness in sport-specific actions

## 1. Introduction

Sports performance generally includes unilateral or bilateral motor tasks that may result in differences between limbs in terms of force and power output [1]. Inter-limb asymmetry—defined as the imbalance in performance between limbs—is a common phenomenon, which particularly characterizes athletes practicing unilateral sports [2]. Several factors were found to explain this asymmetry such as training routines that emphasize unilateral movements, inducing strength imbalances, altered movement patterns, and motor learning adaptations that favor the dominant limb [3]. These adjustments can result in enhanced muscle strength, flexibility, and coordination on the dominant limb, although the non-dominant side may continue to be undeveloped [3]. From a neuromuscular point of view, lateral dominance is considered to represent the functional specialization of the brain’s left or right hemispheres [4]. Moreover, in addition to training factors, lateral dominance is influenced by contextual factors, task complexity, gender, genetics, and training factors [3].

Although some level of asymmetry is acceptable in athletes, values greater than 15% between limbs can negatively impact performance and increase the risk of injury [2]. Bishop, et al. [5], systematically reviewing the literature related to inter-limb asymmetries and their effects on physical performance, found that asymmetries in strength appeared to negatively affect performance activities including change of direction (COD), jumping, and sport-specific skills such as kicking accuracy. Instead, for jumping-based asymmetries, they found varied associations between jumps (vertical and horizontal) and CODS performance [5]. In a recent systematic review with meta-analysis [6], inter-limb asymmetries, quantified through unilateral jumping performance, were found to negatively impact COD and sprinting, but not jumping performance, in cohorts of athletes, supporting the realization that asymmetry is task-dependent and the need for multiple assessments to provide a broad profile of the athlete’s asymmetry. Additionally, Buoite et al. [1] found that muscle asymmetries in male soccer players were associated with performance metrics such as the countermovement jump (CMJ) and tensiomyography, which could influence athletic performance and injury prevention strategies.

As well, the concept of bilateral index (BLI)—which determines whether force generated bilaterally is less than or larger than the sum of unilateral forces— also varies in its effect depending on the type of sport. Specifically, Železnik, et al. [7] reported that bilateral deficit (BLD) can induce positive effect in tennis, basketball, and volleyball, while it is undesirable in other sports like judo.

The implications of asymmetry and bilateral indexes are less well understood in combat sports. While taekwondo research has shown a strong correlation between body composition asymmetries and performance in high-intensity kick tests [i.e., the frequency speed of kick test (FSKT)] [8], judo studies have found that bilateral asymmetry has a negative impact on performance in the Special Judo Fitness Test (SJFT) [9]. For BLI, studies investigating its relationship with performance in combat sports are limited to judo [10,11]. Negative correlations were observed between bilateral deficit (BLD) in CMJ performance metrics and SJFT performance [10]. Conversely, the BLI in the maximal handgrip strength test was not found to be related to SJFT performance in judo athletes [11].

Taekwondo is a full-contact open combat between two athletes that generates the intermittency of the activity in which high-intensity actions (kicks and punches), low-intensity actions (such as skipping), and pauses alternate [12,13]. A wide variety of unilateral technical actions can be observed in current taekwondo combat [14,15]. In addition, athletes must move faster during the combat to keep up with the average match pace imposed by the opponents [16,17], and agility is required to quickly execute technical-tactical movements in multidirectional planes [18]. Thus, the presence of inter-limb asymmetry in power performance and sport-specific anaerobic performance could potentially compromise the athlete’s competition performance considering the relationship between the ability to repeat short high-intensity efforts and scoring actions. In addition, it could lead to an increased risk of injury as taekwondo training largely assumes repetition of typical unilateral and multidirectional technical-tactical actions. In taekwondo, both asymmetry and bilateral indexes have never been investigated although its relationship to performance in other sports contexts is well documented [9,19,20,21].

In this sense, the aim of this study was to investigate the influence of inter-limb asymmetry and bilateral strength deficit in power performance, assessed by squat jump (SJ) and countermovement jump (CMJ), on sport-specific anaerobic performance, by performing the FSKT, in high-level taekwondo athletes. We hypothesized that anaerobic test performance, as measured by both ten seconds frequency speed of kick test (FSKT_10s_) and its multiple version (FSKT_mult_), could be affected negatively by both asymmetry and bilateral indexes measured by jumping tests.

## 2. Materials and Methods

### 2.1. Study Design

This is a cross-sectional study design in which taekwondo athletes were assessed using jumping and technique-specific tests to assess the asymmetry and bilateral (BLI) indexes and their relationship with anaerobic-specific taekwondo performance as assessed using the frequency speed of kick test (FSKT).

### 2.2. Participants

An a priori power analysis (G*Power 3.1.9.7; Heinrich Heine University in Düsseldorf, Germany) indicated that a total sample of 29 subjects would be required with the following settings: bivariate normal model test, two-tailed, power of 0.80, α-value of 0.05, and a correlation of r = 0.50. Forty-eight taekwondo athletes (24 males and 24 females; mean ± SD, age: 19.8 ± 2.1 years, body mass: 62.2 ± 9.4 kg, height: 179.3 ± 9.5 cm, training experience: 8 ± 1 years) volunteered to participate in the present study. To be eligible, athletes should met the following inclusion criteria: (i) they were training four to five times per week, (ii) they were competing regularly during the last 2 years, (iii) athletes were asked to follow the same diet, avoid strenuous exercises, and refrain from caffeine consumption (in drinks and supplements) or any other ergogenic resources during the 48 h before each session, (iv) athletes were not under any weight-loss protocol during the period of experimentation, and (v) for female athletes, only those who were in their follicular phase of the menstrual cycle with a regular cycle duration, defined as a variation lower than 3 days in the range of their menstrual cycles’ length [22] for the previous 2 months, were included. After a detailed explanation of the study’s aims and risks, both parents and athletes provided written informed consent. The study was conducted in accordance with the latest Declaration of Helsinki [23] for human experimentation, followed the ethical standards outlined in the *Publication Manual of the American Psychological Association* [24] and was approved by the Local Research Ethics Committee [Comité de Protection des Personnes SUD (CPP SUD) N^o^. 0332/2021] before data collection.

### 2.3. Procedures

**Figure 1:**
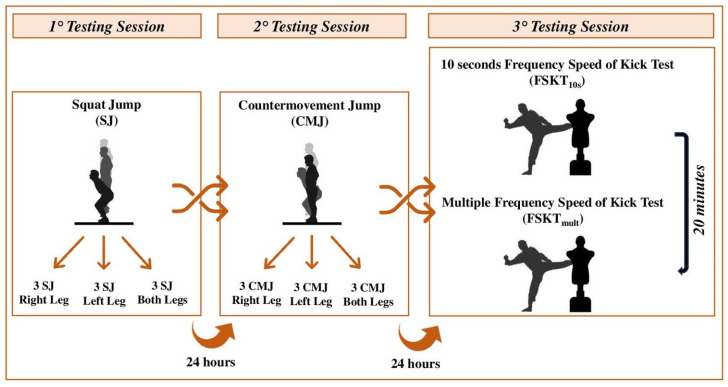
Schematic representation of the study design.

Athletes were well familiarized with the testing procedures (72 h before starting the experiment) and all assessments were conducted at the end of season, at the same time of day as the training sessions (18:00–20:00 h) to avoid any diurnal variation in the performance. Athletes performed randomly, in separate sessions, the squat jump (SJ), countermovement jump (CMJ) tests using the right, left, and both legs, the 10 s frequency of speed kick test (FSKT_10s_), and its multiple version (FSKT_mult_). Before each testing session, athletes performed 10 min of a standardized warm-up session consisting of light runs followed by dynamic stretching and submaximal jumps/technical executions for jumping/kicking testing sessions, respectively (Figure 1).

### 2.4. Measures

#### 2.4.1. Squat Jump Test

From a stationary, semi-squatting position on the right (SJ^R^), left (SJ^L^), or both legs (SJ^BL^), athletes jumped as maximum as possible without arm swing [25] using an infrared jump system (Optojump Next version 1.6.10, Microgate, Bolzano, Italy). Three tests were performed for each mode with 45 s as recovery interval between trials, and the best performance was retained for analysis. The intra-class correlation coefficient (ICC) for test–retest trial with the right, left, and both legs for the present study was 0.82, 0.84, and 0.93, respectively. The coefficient of variation (CV) was 7.42, 6.21, and 4.75%, respectively.

#### 2.4.2. Countermovement Jump Test

From a standing position on the right (CMJ^R^), left (CMJ^L^), or both legs (CMJ^BL^), athletes performed a quick downward movement by flexing the knees and hips immediately followed by a rapid extension of these joints while keeping their hands on their waists. No lower limb flexion or arm swing in the upward phase was allowed [25,26]. The CMJ test was performed using an infrared jump system (Optojump Next version 1.6.10, Microgate, Bolzano, Italy). Three tests were performed for each mode with 45 s as the recovery interval between trials, and the best performance was retained for analysis. The ICC for test–retest trial with the right, left, and both legs for the present study was 0.83, 0.79, and 0.91, respectively. Moreover, CV was 5.86, 5.27, and 7.33%, respectively.

#### 2.4.3. Ten s Frequency Speed of Kick Test

The test consisted of performing the maximum number of bandal-chagi, executed against a bag at the trunk height by alternating legs for ten seconds [27,28]. The ICC and CV for test–retest trial for the present study were 0.90 and 7.81%, respectively.

#### 2.4.4. Multiple Frequency Speed of Kick Test

Each athlete performed five sets of FSKT_10s_ alternating both legs, with a ten-second rest interval between repetitions [27,28], and the total number of techniques was used for subsequent analysis. The ICC and CV for test–retest trial for the present study were 0.88 and 8.02%, respectively.

#### 2.4.5. Interlimb Asymmetry and Bilateral Indexes from Jumping Tests

Interlimb asymmetry was quantified as the percentage of difference between the stronger and weaker limb using Equation (1) previously proposed [29]:

Interlimb asymmetry (%) = (Stronger limb − Weaker limb)/Stronger limb × 100 (1)

The bilateral index (BLI), expressed as a percentage, was calculated using Equation (2) as previously proposed [30]:

BLI (%) = 1 − (bilateral/(right + left)) × 100 (2)

A positive percentage score indicates the presence of a bilateral facilitation (BLF) where the bilateral performance is superior to the summed unilateral performances, whereas a negative percentage is indicative of a bilateral deficit (BLD) [31].

### 2.5. Statistical Analyses

Statistical analysis was performed using SPSS 20.0 statistical software (SPSS Inc., Chicago, IL, USA). The normality of datasets was checked and confirmed using the Shapiro–Wilk test. Spearman’s correlation coefficient (ρ) was used between FSKT_10s_ performance and the asymmetry indexes from CMJ and SJ, and between FSKT_mult_ and the asymmetry and bilateral strength indexes from CMJ and SJ. Pearson’s product-moment correlation coefficient (*r*) was used to examine relationships between FSKT_10s_ performance and bilateral strength indexes from CMJ and SJ. Magnitude of correlation was determined as trivial when *r* < 0.1, low 0.1–0.3, moderate 0.3–0.5, large 0.5–0.7, very large 0.7–0.9, nearly perfect > 0.9, or perfect = 1 [32]. Multiple regression analyses were used to establish the relationship between the dependent variables (FSKT_10s_ and FSKT_mult_) and the independent variables (SJ and CMJ asymmetry indexes and SJ and CMJ bilateral strength indexes), based on collinearity principles [33]. The correlation coefficient value was determined and the coefficient of determination (*R^2^*) was calculated to determine the proportion of the variability in the independent variables explained by the dependent variables. The standard error of estimate (SEE) of each equation is also presented. Statistical significance was set at *p* < 0.05.

## 3. Results

**Figure 2:**
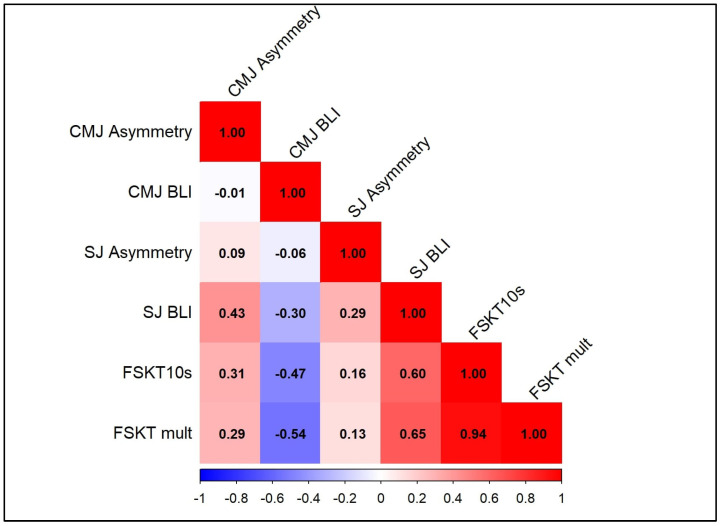
Correlations between technical performance, asymmetry, and bilateral indexes (BLIs) during squat jump (SJ) and countermovement jump (CMJ) tests. FSKT: frequency speed of kick test.

Figure 2 presents the correlations between technical performance, asymmetry, and bilateral indexes during jumping tests.

Regression analyses showed that ten seconds frequency speed of kick test (FSKT_10s_) performance could be partially predicted from CMJ and SJ bilateral indexes using the following Equation (3):

FSKT_10s_ (n°) = 19.16 − 0.109 (CMJ-BLI in cm) + 0.081 (SJ-BLI in cm), *r* = 0.677, *R*^2^ = 0.459, SEE = 1.909, and *p* < 0.001 (3)

Regression analyses showed that multiple sets of frequency speed of kick test (FSKT_mult_) performance could be partially predicted from CMJ and SJ bilateral indexes using the following Equation (4):

FSKT_mult_ (n°) = 93.008 − 0.587 (CMJ-BLI in cm) + 0.428 (SJ-BLI in cm), *r* = 0.747, *R*^2^ = 0.557, SEE = 7.966, and *p* < 0.001 (4)

## 4. Discussion

This study explored the relationship between taekwondo-specific tests [ten seconds of frequency speed of kick test (FSKT_10s_) and its multiple sets version (FSKT_mult_)], interlimb asymmetry, and bilateral strength deficit (BLD) assessed by vertical jump tests (i.e., CMJ and SJ) in high-level taekwondo athletes. The main findings showed that CMJ and SJ BLI explained 45.9% of the variance of the FSKT_10s_ performance, whilst they explained 55.7% of the variance of FSKT_mult_ performance confirming the hypothesis of the present study.

The FSKT is an important test, which has been explored over the last few years, considering performance in taekwondo based on construct classificatory tables [34], reliability, sensitivity, and construct validity [27], group discrimination between competitive levels [35], post-activation potentiation conditioning [36,37], and correlated with performance in the CMJ [38]. Based on these aspects, the FSKT is an excellent alternative to assess the anaerobic fitness of taekwondo athletes in an intermittent and sport-specific mode. The FSKT (10 s and multiple) performance could be partially predicted from CMJ and SJ bilateral strength indexes. The neural responses, reflecting the imbalance between unilateral and bilateral performance, can be explained by the CMJ and SJ tests. This relationship is evident in the specific equations derived from the FSKT_mult_ and FSKT_10s_ performance.

The use of vertical jump tests as an indicator of interlimb asymmetry, based on the jump height metric [39], is a good alternative to assess the imbalance between limbs in high-level athletes [5,40], considering different sports modalities [5], and more recently, combat sports athletes [9,41]. The relationship between FSKT_10s_ and FSKT_mult_ and interlimb asymmetry demonstrated that the high number of kicks performed at high intensity is related to the high values of interlimb asymmetry assessed by the CMJ and SJ tests. This suggests that the repetitive nature of kicking with one leg can lead to greater strength and performance discrepancies between the dominant and non-dominant legs.

It is well known that due to the nature of the taekwondo modality, athletes are expected to present structural and functional asymmetries due to long-term specialized training since the kicks performed during specific tasks are performed unilaterally [40,42]. In this sense, the aspect of dominance significantly influences taekwondo performance giving that athletes normally have one side stronger than the other and consequently superior strength and power on the dominant kicking side.

From a psychopathological perspective, various factors can contribute to asymmetry and bilateral deficit in motor performance by disrupting the brain’s ability to coordinate and control movement symmetrically [43]. Neurological conditions, such as those resulting from previous injuries, can impair the brain’s control over one side of the body more than the other, leading to imbalances in tasks that require bilateral coordination [44]. This imbalance might manifest in taekwondo as uneven kicking power or poor balance when performing moves that rely on both sides. Additionally, psychological factors, including anxiety or stress, can also affect motor performance by increasing muscle tension or disrupting fluid movement [45], which may result in uneven performance between the left and right sides of the body. Furthermore, cognitive dysfunctions related to conditions like depression can impact executive functions such as focus, task-switching, and decision-making [46] which are crucial in high-intensity sports like taekwondo. Consequently, these psychopathological factors can provide valuable insight into the correlation between asymmetry and bilateral deficit in jumping performance and specific performance in taekwondo by influencing both the motor and cognitive systems essential for executing symmetric movements.

In specific performance, Liu et al. [42] reported that during a double roundhouse kick in sub-elite taekwondo athletes, there was a difference between dominant and non-dominant side in term of maximum knee flexion angle and the peak linear velocity of attack of the foot in the vertical hitting direction for the attacking leg and the peak hip extension moment for the supporting leg. Furthermore, in terms of strength, studies have shown significant differences between preferred and non-preferred legs in female taekwondo athletes [47] and in a mixed sample of athletes [48]. Similarly, Harbili et al. [49] reported that, in elite male and female taekwondo athletes, isometric hamstring strength was greater in the dominant leg by 15% for male athletes and 11% for female athletes aligning with the expected outcomes of unilateral training. However, the same study reported no significant differences in isokinetic and isometric knee strength between the dominant and non-dominant legs in male and female athletes reporting the absence of bilateral knee strength asymmetry.

Conversely, in terms of body composition, Ojeda-Aravena et al. [8] showed a negative relationship between body composition asymmetry and performance in FSKT; however, the values of body composition asymmetry were very low and did not reflect the asymmetry observed in unilateral physical tests in this study for example.

The bilateral index of strength measures force production in both unilateral and bilateral performance [7] across various tasks, such as isometric leg extension [50], vertical jump tests [10], and isometric handgrip strength tests [11]. Negative values in these tests indicate a bilateral deficit, whilst positive values suggest neuromuscular facilitation [7]. In this study, a negative relationship was found between the bilateral index and FSKT performance in both protocols. Specifically, a high negative bilateral index in CMJ negatively impacted FSKT performance. Practically, this suggests that taekwondo athletes need to be strong, stable, and powerful in both limbs to maximize their chances of successful defense and attacks during taekwondo-specific actions [42]. Therefore, it may be advantageous to reduce the bilateral deficit (i.e., improve bilateral jumping relative to unilateral jumping) to optimize taekwondo-specific performance, like the findings observed recently in judo [10].

Based on the results of the present study, coaches and strength and conditioning trainers should consider incorporating unilateral training exercises focusing not only to enhance the strength of the dominant leg but also to address the non-dominant leg to reduce interlimb asymmetry and thus maintain a balanced athletic profile and optimize overall specific performance. Additionally, conditioning training programs should be oriented to address the bilateral strength deficit by using exercises that are performed bilaterally (e.g., implementing bilateral strength exercises and ensuring that both legs are equally engaged and capable of generating force during training) irrespective of unilateral movements. This can improve the symmetry in terms of force production across both legs which may enhance performance in taekwondo-specific movements and may potentially enhance their kicking power and overall effectiveness.

### Limitations and Future Perspectives

Although our study offers novel information, several limitations must be acknowledged. Firstly, during CMJ and SJ tests, only jump height was used to calculate asymmetry and bilateral indexes. In this sense, other metrics (e.g., power, velocity) might be measured to increase the amount of relevant information and insights that can be derived from the tests. Secondly, Fox et al. [6] indicated that asymmetry is task-dependent and there is a need for multiple assessments to provide a broad profile of the athlete’s asymmetry. Therefore, it might be important to expand the present investigation by also using another taekwondo sport-specific test, such as the TSAT, as during combat, agility is required to quickly execute technical-tactical movements in multidirectional planes [18]. Finally, a limitation in studying asymmetry and bilateral deficit in this study is the lack of taking sex factor into consideration. Sex plays a crucial role in muscle properties, including differences in muscle mass, strength, and endurance between males and females. These variations may influence the degree of asymmetry and bilateral deficit observed in athletes. Future studies can be conducted using electromyography and tensiomyography to explore the neuromuscular mechanisms underlying the observed relationships between jump asymmetries/bilateral indexes and anaerobic kicking performance in taekwondo athletes. This may help to explore whether this difference between the dominant and non-dominant leg during jumping is due to neural mechanisms (e.g., muscle activation timing, recruitment patterns) or contractile muscles’ characteristics (e.g., stiffness, contraction time, and relaxation time).

## 5. Conclusions

The present study showed that performance in FSKT_10s_ was positively correlated with CMJ asymmetry and SJ BLI and negatively correlated with CMJ BLI, and that FSKT_mult_ was positively correlated with SJ BLI, and negatively correlated with CMJ BLI. In addition, CMJ and SJ BLI explained 45.9% of the variance of the FSKT_10s_ performance, whilst they explained 55.7% of the variance of FSKT_mult_ performance. These results highlight the importance of managing both interlimb asymmetry and bilateral indexes to achieve better performance.

## Data Availability

The datasets presented in this article are not readily available [the data are part of an ongoing study]. Requests to access the datasets should be directed to the corresponding author.

## Abbreviations

BLI	Bilateral index
SJ	Squat jump
CMJ	Countermovement jump
FSKT10s	Ten s Frequency Speed of Kick Test
FSKTmult	Multiple Frequency Speed of Kick Test
FSKT	Frequency Speed of Kick Test
TSAT	Taekwondo-Specific Agility Test
COD	Change of direction
SJ^R^	Squat jump with right leg
SJ^L^	Squat jump with left leg
SJ^BL^	Squat jump with both legs
CMJ^R^	Countermovement jump with right leg
CMJ^L^	Countermovement jump with left leg
CMJ^BL^	Countermovement jump with both legs
ICC	Intra-class correlation coefficient
CV	Coefficient of variation
BLF	Bilateral facilitation
BLD	Bilateral deficit
ρ	Spearman’s correlation coefficient
R^2^	Coefficient of determination
